# Sociodemographic Determinants for Oral Health Risk Profiles

**DOI:** 10.1155/2010/938936

**Published:** 2010-02-04

**Authors:** J. Vanobbergen, L. De Visschere, M. Daems, A. Ceuppens, J. Van Emelen

**Affiliations:** ^1^Community Dentistry and Oral Public Health, Dental School, Ghent University, 9000 Ghent, Belgium; ^2^Division Research & Innovation, National Union of Independent Mutual Health Insurance Service, 1150 Brussels, Belgium

## Abstract

The present study aimed to explore the association between caries risk profiles and different sociodemographic factors . The study sample (*n* = 104) was randomly selected within an urban population in Flanders, Belgium. Caries risk was assessed by anamnesis, clinical examination, salivary tests, and a questionnaire. Age, gender, and socio-economic status were extracted from social insurance data files. Social indicators were “occupational status,” “being entitled to the increased allowance for health care interventions” and having access to the “Maximum Bill” (MAF), initiatives undertaken to protect deprived families. In the bivariate analysis there were significant differences in risk profiles between occupational groups (*P* < .001), between entitled and non-entitled individuals to the increased allowance (*P* = .02), and between access or no-access to the MAF (*P* < .01). The multiple logistic model showed a significantly higher chance of being in the low risk group for individuals with no-access to the MAF compared to those with access (OR:14.33–95% C.I. 2.14–95.84).

## 1. Introduction

Taking into account new insights in the management of diseases, a patient-centred holistic approach is recommended. This involves that care providers should respect patients' prospects, concerns, preferences, wants and needs, and solicit patients' input into decisions [1]. 

This person-centred approach is very important in preventive care and is directed to increase patients' knowledge and beliefs, self-regulation skills and abilities, and social facilitation [2]. An initial assessment of these factors together with biological predictors of a potential disease will be part of new preventive health management strategies.

In order to plan appropriate, patient-centred caries management in oral health care, frameworks are elaborated which the dental team can use to bring together key elements of information about patients and patients' teeth. Recently “risk assessment” and “early detection” were focused [3–5]. “Risk assessment” aims to detect unfavourable factors before the initiation of the disease. It is the process of quantifying the probability of a harmful effect to individuals or populations from certain human activities or from unfavourable environmental factors. “Early detection” aims to detect any disease process in a very early stage.

Risk assessment is part of a primary prevention strategy, early detection is part of the secondary prevention.

Caries management by risk assessment (CAMBRA) coupled with early detection and a quick and effective response can be seen as one of the best and cost-efficient ways of dealing with one of the most prevalent oral health problems, caries. This “medical model,” where the etiologic disease-driving agents are balanced against protective factors, and integrated in a risk assessment model, offers the possibility of patient-centred disease prevention and management before there is irreversible damage done to the teeth [6]. 

The rational for a caries risk assessment management in industrialized Western countries is as follows.

- A rather low incidence of the disease in the general population justifying the efforts and costs to identify high-risk groups. In the late 70s the incidence of caries was very high and omnipresent in all age groups. In contrast, today caries prevalence and incidence decreased and are concentrated in 20% of the population. An attempt to identify individuals and groups expected to be at high-risk seems sensible.
- Risk assessment as a screening activity without followup and an adapted targeted prevention is useless. On the other hand, when linked to an explicit strategy of targeted preventive care to well-defined and -identified risk groups, it becomes very useful, even compelling.
- The match of preventive care to the individual risk profile of specific individuals or groups avoids wastage of already scarce resources.

Caries risk assessment combines an assessment of disease indicators and risk factors. A small number of key disease indicators and risk factors determine whether the individual is at low, moderate, or high-risk.

Risk factors can be biological, behavioural, or socioeconomic contributors to the caries disease process that can be modified as part of the treatment plan. If the disease is currently active, or if there is the future risk of progression of dental caries, intervention appropriate to the risk status is required to correct the caries imbalance before cavitation occurs [6].

The most difficult parameters to be modified are the socioeconomic contributors. Oral health risk profiles may be unevenly spread over the various social groups in the population. Insight of the risk profile of social vulnerable groups is an interesting item to pay attention to in the implementation of caries management by risk assessment. 

Risk-based prevention programmes can be effective in further reducing dental caries in a low-caries community; this is demonstrated in previous research, especially targeting very young children [7]. Further research, exploring the perspectives of public health and targeting socially deprived groups, can contribute to this further reduction.

## 2. Objective

The aim of the present study was to explore the association between risk profile for dental caries and different sociodemographic factors on an individual level.

## 3. Material and Methods

The study sample (*n* = 1000) was randomly selected, after stratification by age, within the population of a metropolitan area in Flanders, Belgium: Ghent and surroundings. Five age groups were defined. Invitations to participate were sent in four consecutive quarters, starting in November 2007 and ending in April 2008. 

Data from clinical examination, salivary tests, health anamnesis, and an oral health habits questionnaire were used to assess oral health risk. In particular, caries risk was assessed including

- past caries experience (clinical examination),
- assessment of the general health (mainly diabetes, epilepsy, polypharmacy, and smoking habits) (health anamnesis),
- diet: intake of nutrients with high sugar concentration and frequency (number of meals and between-meals) (questionnaire),
- oral hygiene: frequency of tooth brushing (questionnaire),
- quantity of clinical observable dental plaque (clinical examination),
- fluoride programme (questionnaire),
- saliva: flow and buffer capacity (salivary tests),
- risk enhancing dental patterns: crowding, exposed root surfaces, and ill-fitting restorations (clinical examination).

Three examiners participated in the oral examinations. A calibration for the diagnostic criteria of caries was performed on 43 teeth, registered within 21 clinical cases. The inter-examiner reliability was high, with weighted kappa values being 0,97, 0,93 and 0,92 for the respective examiners.

The oral health habits questionnaire was previously validated (content validity) and tested for reliability in a test-retest procedure with 12 participants. 

Analyses were performed taking the risk profile as a dependent variable. Risk profile was calculated as a percentage and reduced to a categorical variable in the inferential analyses. Three risk levels have been defined: low (25% or less), moderate (between 25% and 75%) and high (75% and higher). 

Age, gender, and socioeconomic status were used as independent variables. They were extracted from social insurance data files. Social indicators were “occupational status”, “being entitled to the increased allowance for health care interventions” and “having access to the mechanism known as the Maximum Bill (MAF)”. The two last mentioned initiatives were undertaken to improve access to the health care system and to protect deprived families from large expenses for health care. 

Nonparametric bivariate analyses by means of nonparametric tests (Mann-Whitney U and Kruskal Wallis for 2 or more independent groups, resp.), and multiple logistic regression were performed to estimate the contribution of the independent risk indicators. The analyses were carried out using SAS statistical program. The level of significance was set at 0.05.

## 4. Results

The response rate was low with 104 out of 1000 invited participants accepting the invitation and presenting themselves at the dental clinic for the oral examination. The third quarter presented the lowest response rate while for the first call the response rate was the highest (12%). There was little difference between responders and non-responders in terms of gender, age and social indicators. There was a small over representation of participants of forty and a small under representation of self-employed in the responder group.

### 4.1. Explorative Data Analysis

**Figure 1:**
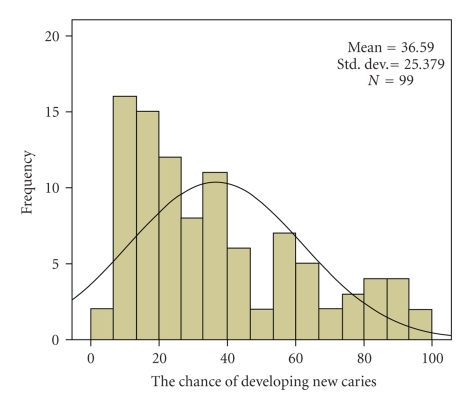
The chance of developing new caries expressed in a percentage.

The average chance of developing new caries, calculated on the basis of the risk profile as it was described in Section 3 is shown in Figure 1.

The overall average chance of developing new caries was 36,6%. A rather equal spread was found between the different risk factors diet (9.3%), oral hygiene and plaque amount (11.3%), fluoride program and saliva properties (10.1%), and past caries experience and related diseases (5.9%).

The distribution of the chance of developing new caries was left-skewed. 45,5% of participants belonged to the “low risk” group, meaning that they have less than 25% chance of developing new caries, 44,4% belonged to the “moderate risk” group and 10,1% belonged to the “high risk” group, which has a mean chance of 87% of developing new caries.

### 4.2. Inferential Analysis

In the bivariate analysis (Table 1) risk profiles were not significantly different between age groups and between males and females. All social variables showed strong and significant links with the risk profile. There were significant differences between occupational groups (*P* < .001), between entitled and non-entitled individuals to the increased allowance (*P* = .02), and between access or no-access to the MAF (*P* < .01). Participants from lower social classes showed a significantly higher mean risk profile for developing new caries. 

The most important factors related to dental caries in this group were an inadequate fluoride program (mainly frequency of tooth brushing with a fluoride toothpaste), and insufficient oral hygiene (plaque amount) (Table 2).

The multiple model (Table 3) showed that the chance of being in the low risk group for individuals with no-access to the MAF was 14 times higher compared to the individuals with access to the MAF (OR:14.33–95% C.I. 2.14–95.84).

## 5. Discussion

The findings indicate that risk-based prevention can be correctly targeted to socially vulnerable groups within the community. A stepwise use of risk assessment tools can be very helpful to further decrease caries prevalence in different age groups. This complements the findings reported in earlier research targeting the group of very young children [7]. A first step will be to identify risk groups within the community, the second to identify high-risk individuals within these high-risk groups and finally, to identify risk profiles. 

Within the present study lifestyle related factors have been identified as important risk factors for caries in high-risk groups, in particular in socially vulnerable high-risk groups. Fluoride programmes, assessed by the frequency of tooth brushing with a fluoride toothpaste and intake of fluoride supplements, have an important impact on the risk profile of these groups. Oral hygiene, expressed as the amount of dental plaque, seems to have an important negative impact on caries risk profiles of socially vulnerable groups. 

In risk-based prevention targeting social vulnerable high-risk groups, these lifestyle related factors will be an important feature. Effectiveness of health education, dealing with lifestyle related factors, has been demonstrated in low socioeconomic families [8], but extra efforts will have to be done to implement strategies for changing oral health behaviour in order to have a long-term impact on risk profiles. Patient-dentist communication will be extremely important. The usefulness of additional therapeutic contacts via a combination of telephone coaching, mobile phone Short Message Service or even electronic mail, as introduced in other health care settings [8], has to be considered. 

Of course it should be noted that these data are based on a rather small sample.

The response rate was low. This is a weakness of the study. This is typical for this kind of surveys with people randomly invited to participate and relying only on their own initiative to make an appointment in the dental clinic. Since the profile of responders and non-responders did not differ significantly the effect of the low response rate can be considered limited. Further longitudinal research will be opened to explore the clinical and economic effectiveness of risk-based prevention programmes, particularly in identified high-risk groups, including extra communication tools to increase patient adherence.

## 6. Conclusion

All social variables showed strong and significant links with the caries risk profile. For each social category a gradation has been observed between the three different oral health risk levels. Stepwise risk-based prevention opens opportunities to further decrease caries prevalence in low-prevalence communities.

## Figures and Tables

**Table 1:** Cross-tabulation for different sociodemographic variables and the three caries risk levels (Mann-Whitney U and Kruskal Wallis for 2 or more independent groups, resp.).

	Low risk	Moderate risk	High risk	*P*-value
Age				NS
<12 years (*n* = 6)	33,3%	66,7%	0%	
Young (*n* = 19)	47,4%	26,3%	26,3%	
Adults (*n* = 54)	46,3%	46,3%	7,4%	
60+ (*n* = 20)	45%	50%	5%	
Gender				NS
Male (*n* = 42)	40,5%	52,4%	7,1%	
Female (*n* = 57)	49,1%	38,6%	12,3%	
SES				<,0001
Worker (*n* = 21)	23,8%	61,9%	14,3%	
Employee (*n* = 30)	66,7%	26,7%	6,7%	
Managerial (*n* = 11)	81,8%	18,2%	0%	
Self-employed (*n* = 7)	14,3%	57,1%	28,6%	
Others (*n* = 2)	0%	0%	100%	
Increased allowance				0,02
No (*n* = 80)	52,5%	40%	7,5%	
Yes (*n* = 12)	25%	41,7%	33,3%	
MAF Family (Maximum Bill)				<,01
No (*n* = 82)	52,4%	40,2%	7,3%	
Yes (*n* = 10)	20%	40%	40%	

**Table 2:** Differences in mean risk profiles and components for different social groups.

	Chance of developing new caries	Fluoride programme	Amount of dental plaque	Diet
Access to MAF*	54.20%	22.40%	15.20%	10.50%
No access to MAF	33.27%	8.50%	10.50%	8.50%
*P*-value	.01	.003	.05	.5

*MAF: Maximum bill, a mechanism to protect deprived families from large expenses for health care.

**Table 3:** Odds ratio for the chance of being in the low caries risk group (adjusted for age, gender, and occupational status).

	Odds ratio	95% CI	*P*-value
Access to the Maximum Bill	1		
No access to the Maximum Bill	14.33	2.14–95.84	.006
